# Measuring postnatal care contacts for mothers and newborns: An analysis of data from the MICS and DHS surveys

**DOI:** 10.7189/jogh.07.020502

**Published:** 2017-12

**Authors:** Agbessi Amouzou, Vrinda Mehra, Liliana Carvajal–Aguirre, Shane M. Khan, Deborah Sitrin, Lara ME Vaz

**Affiliations:** 1 Johns Hopkins Bloomberg School of Public Health, Baltimore, Maryland, USA; 2 Data and Analytics Section, Division of Data, Research and Policy, UNICEF, New York, USA; 3 JHPIEGO, Baltimore, Maryland, USA; 4 Save the Children, Washington, D.C., USA

## Abstract

**Background:**

The postnatal period represents a vulnerable phase for mothers and newborns where both face increased risk of morbidity and death. WHO recommends postnatal care (PNC) for mothers and newborns to include a first contact within 24 hours following the birth of the child. However, measuring coverage of PNC in household surveys has been variable over time. The two largest household survey programs in low and middle–income countries, the UNICEF–supported Multiple Indicator Cluster Surveys (MICS) and USAID–funded Demographic and Health Surveys (DHS), now include modules that capture these measures. However, the measurement approach is slightly different between the two programs. We attempt to assess the possible measurement differences that might affect comparability of coverage measures.

**Methods:**

We first review the standard questionnaires of the two survey programs to compare approaches to collecting data on postnatal contacts for mothers and newborns. We then illustrate how the approaches used can affect PNC coverage estimates by analysing data from four countries; Bangladesh, Ghana, Kygyz Republic, and Nepal, with both MICS and DHS between 2010–2015.

**Results:**

We found that tools implemented todate by MICS and DHS (up to MICS round 5 and up to DHS phase 6) have collected PNC information in different ways. While MICS dedicated a full module to PNC and distinguishes immediate vs later PNC, DHS implemented a more blended module of pregnancy and postnatal and did not systematically distinguish those phases. The two survey programs differred in the way questions on postnatal care for mothers and newbors were framed. Subsequently, MICS and DHS surveys followed different methodological approach to compute the global indicator of postnatal contacts for mothers and newborns within two days following delivery. Regardless of the place of delivery, MICS estimates for postnatal contacts for mothers and newbors appeared consistently higher than those reported in DHS. The difference was however, far more pronounced in case of newborns.

**Conclusions:**

Difference in questionnaires and the methodology adopted to measure PNC have created comparability issues in the coverage levels. Harmonization of survey instruments on postnatal contacts will allow comparable and better assessment of coverage levels and trends.

The postnatal period, days and weeks following childbirth, is a vulnerable phase in the lives of mothers and newborns. Deaths within the first month of life represent 45% of all under–five deaths [1] and of these, far too many occur within the first week of birth. In 2015, nearly one million neonatal deaths occurred on the day of birth and close to two million newborns died in the first week of life [2]. Women too face an increased risk of morbidity and death after delivery. Maternal complications such as bleeding and sepsis following childbirth are responsible for over one–third of the maternal deaths worldwide [3]. To support mothers and newborns during this critical phase, postnatal care (PNC) was identified as a critical need by the World Health Organization (WHO) in 1997 [4]. In 2004, this recommendation was again highlighted in WHO’s guidelines for pregnancy, childbirth, postnatal and newborn care [5]. Postnatal care guidelines were recently reviewed to recommend the number, timing and content of postnatal care contacts. WHO recommends the first postnatal contact within 24 hours of birth, followed by three additional contacts on day 3, between days 7–14 and six weeks after birth. In case of facility based deliveries, newborns should receive an immediate check at birth, full clinical assessment around one hour after birth and before discharge [6].

Recent research estimates that increased coverage of postnatal interventions, along with quality interventions from preconception to birth can save 1.9 million neonatal deaths annually. [7]. Postnatal care home visit from a trained provider within two days of delivery can lead to 30–40% reduction in neonatal mortality [8,9]. Given the significance of postnatal period and the effectiveness of postnatal care, it was essential that its coverage is measured and monitored at global and country level. In 2010, the countdown to 2015 called on the importance of developing and expanding the measurement and availability of data on PNC [10]. More recently with the launch of the United Nations Global Strategy for Women’s, Children’s and Adolescent’s Health [11] and the Lancet Every Newborn Series in 2014 [12] the international community has agreed on new frameworks for global monitoring of MNCH targets. These recent frameworks which include Every Newborn Action Plan (ENAP) and Ending Preventable Maternal Mortality (EPMM) have included and prioritized postnatal care as a core coverage monitoring indicator.[11,13,14].

Although there is consensus on the importance of care during this period, the definition and measurement of PNC contacts with the mother and newborn have been a challenge. With regard to definition, an important issue described by the Newborn Technical Working Group deals with timing of postnatal care [15]. There is a lack of consensus among experts as to when the intrapartum period ends and the postnatal period begins. Other studies have analyzed the validity and reliability of respondents’ answers regarding the timing of postnatal health check [16–18]. Timing of postnatal health check has typically ranged from minutes to days. Thus, many of these contacts may be part of the routine intrapartum care rather than distinct postnatal care contacts [15,16]. The confusion in the timing and content of PNC also led to further challenges in the measurement from household surveys. To measure, PNC, it is essential to convey appropriately a clear understanding to the respondent of what interventions are considered PNC, the timing, location (facility or outside facility) and provider of the interventions.

Large–scale, nationally representative household surveys such as the UNICEF–supported Multiple Indicator Cluster Surveys (MICS) [19] and the USAID–funded Demographic and Health Surveys (DHS) [20] now systematically collect data on PNC in their standard tools. Both survey programs report on the global postnatal care indicator for mothers and newborns which is defined as the “postnatal health check for the mother (or newborn) within two days of delivery”. However, there are differences in the survey tools with MICS round four introducing a detailed module on PNC, tested in consultation with the Newborn Technical Working Group [15,21]. A couple of studies have raised the difference in MICS and DHS protocols along with their potential implications on MNCH coverage indicators, and called for greater attention to harmonizing the indicators [15,22]. With regards to PNC, there has not been a systematic assessment comparing the measurement approaches implemented by MICS and DHS, the two largest source of population–based MNCH coverage data in low and middle–income countries, so it has not been clear how questionnaire differences may affect the level and interpretation of PNC coverage.

The aim of the present study is to assess the data on postnatal care of mothers and newborns collected by MICS and DHS and compare PNC measures. In the first part of this paper, we compare the standard questionnaires of MICS and DHS. To illustrate and further study how differences in questionnaires may affect coverage levels, we then review the computation approach of the PNC coverage indicators in four countries with available data from both surveys.

## DATA AND METHODS

### Data

The data for this study come from standard individual women’s questionnaires used in MICS and DHS. The questionnaires were obtained from the website of these survey progams [23,24]. The MICS survey program works in rounds and is currently in its round six. DHS is implemented in phases and is currently in its seventh phase. PNC questions are asked to women age 15–49 years with a last live birth in the recent past, generally the past two to five years. Questions are asked regardless of whether the child is still alive or not.

For the quantitative assessment of data on postnatal care, we first used estimates on postnatal care coverage within two days of delivery for women and children from all available DHS and MICS reports during the period 2010–2015. We then identified six countries that had a MICS and a DHS survey of the 60 countries with DHS and/or MICS during this period. The selection of six countries namely, Bangladesh, Ghana, Kyrgyz Republic, Malawi, Nepal and Zimbabwe was intended to be illustrative rather than representative of countries across the two survey programs. Out of these, we retained four countries because the DHS survey in Malawi and Zimbabwe in 2010 and 2011 respectively, did not collect all the required information on postnatal contacts of mothers and newborns. Survey sample sizes in the four countries examined are included in **Table 1**.

**Table 1:** Countries included in the analysis, data sources and sample sizes

Country	MICS			DHS		
**Year**	**Number of households**	**Number of women (15–49 years)**	**Year**	**Number of households**	**Number of women (15–49 years)**
Bangladesh	2012–2013	55 120	29 599	2014	17 989	18 245*
Ghana	2011	12 150	10 963	2014	12 841	10 963
Kyrgyzstan	2014	7 190	6 995	2011	8 208	8 286
Nepal	2014	13 000	14 936	2011	11 353	12 918

DHS – Demographic and Health Surveys, MICS – Multiple Indicator Cluster Surveys

*Data was collected only on ever married women.

### Statistical analysis

We first described the coverage of postnatal care within two days of delivery for mothers and newborn using all available and consistent data from MICS and DHS reports during the period 2010–2015. Then for each survey program, we reviewed the model questionnaires starting from phase 4 for DHS and round four for MICS, when questions on PNC were first introduced in each programme. We examined the wording of the PNC questions asked to mothers and the reference populations used. We finally compared data collected on postnatal contacts using questionnaires from MICS round five and DHS phase 6 as these survey rounds had quantitative data available at the time of analysis and mapped the algorithm of measurement of postnatal contacts across the 2 survey programs. We could not include data from the latest MICS round six and DHS phase 7 surveys as no databases on these revised tools were available at the time of completion of this analysis. The observed difference in questionnaires was then used to investigate any difference in the PNC indicator values across the two survey programs.

Focusing on the four countries listed above, we then carried out a quantitative description of variables on postnatal contact and timing of health check from MICS and DHS data sets. To investigate sources of differences between surveys, we calculated coverage of any PNC separately for mothers and newborns and for facility and non–facility births, then distinguished “immediate checks” and “postnatal care visits”. Immediate checks refer to women who gave birth in a health facility and who received a check before discharge or to women who gave birth outside a health facility in presence of a birth attendant (health professional or trained birth attendant) and who had a check before the attendant left. A “postnatal care visit” is considered occurring after discharge or after the birth attendant has left or any check for women who gave birth without an attendant. A postnatal health check refers to either of those checks and is accounted for in the measurement of the PNC indicator [25]. We then calculated the global indicator of postnatal care within two days after birth. The global indicator as reported in survey reports was calculated separately for institutional and non–institutional births by consistently excluding postnatal health checks by a relative, family or friends. Our estimates for postnatal care of mothers and newborn differ from the survey report in case of Bangladesh DHS 2014 as the latter reports on postnatal health checks by only medically trained providers among live births in last three years while we follow a standardized approach of assessing postnatal care among live births in two years preceding the survey.

We then used the data collected in these 4 surveys to investigate the distribution of timing of health checks for mothers and newborns. Both survey programs report time of postnatal health checks in units of hours, days or weeks. However, for the purposes of this analysis, timing of postnatal health check was computed and assessed in terms of days, going from 0 to 42 days.

### Ethical review

Data used in this study come from publicly available data which are anonymized and therefore no ethical approval was sought. The Institutions that collected the data are responsible for securing the appropriate ethical approval prior to data collection.

## RESULTS

### PNC Coverage patterns

**Figure 1:**
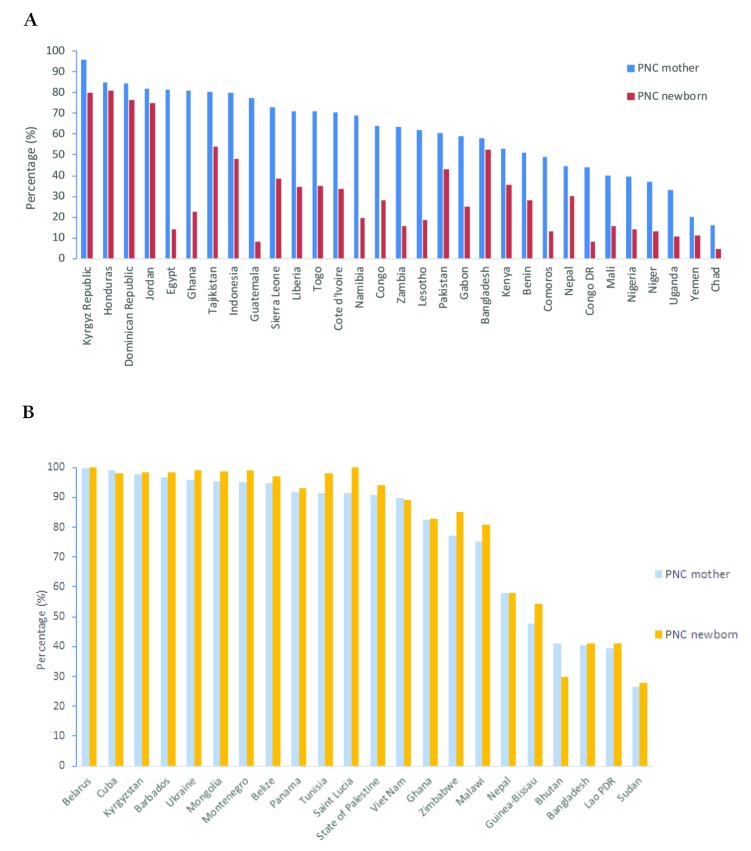
Coverage of postnatal care within two days of delivery for mothers and newborns, Demographic and Health Surveys (DHS) and Multiple Indicator Cluster Surveys (MICS) (2010–2015). **A.** DHS data. **B.** MICS.

**Figure 1** shows the coverage of postnatal care within two days for mothers and newborns, using all available consistent data from MICS and DHS surveys between 2010–2015. The figure compares PNC for mothers and newborns separately for (A) and for MICS (B), and for different set of countries. The figure indicates variable levels of coverage across countries but highlights two important features. On the one hand, for DHS data, coverage levels of PNC appear higher for mothers than for newborns for all countries. On the other hand, for MICS, coverage of PNC appears fairly similar for mothers and for newborns, with newborns appearing to have a slight advantage over mothers in some countries (Tunisia, Saint Lucia, Zimbabwe and Malawi and Guinea–Bissau). The differential patterns between MICS and DHS persist in countries, such as Ghana and Kyrgyz Republic that had both types of surveys within the period examined.

### Measuring PNC in DHS

**Table 2** provides the evolution of introduction of PNC questions in DHS questionnaires by phase, and specific questions introduced. PNC questions were first introduced in phase 4 questionnaire in 1997. These questions were asked only to women who delivered outside a health facility. No attempts were made to measure PNC of newborns. In 2003, a new phase questionnaires were introduced (phase 5) which extended the PNC questions to all women regardless of place of delivery. In addition, questions on PNC for newborn were collected for the first time . However, they were asked only about facility births. From 2008, the phase 6 questionnaires extended PNC questions to all women and newborns, regardless of place of delivery. In addition, effort was made to ensure correct understanding of women’s health check by the respondent by stating during the interview examples of actions that would be considered a health check.

**Table 2:** Overview of PNC data collected in Demographic and Health Surveys (DHS)

	Phase 4 (1997–2003)		Phase 5 (2003–2008)		Phase 6 (2008–2013)		Phase 7 (2013–2018)	
**Facility births**	**All home births**	**Facility births**	**All home births**	**Facility births**	**All home births**	**Facility births**	**All home births**
**Postnatal care: Women**								
Timing of 1st check	X	Yes	Yes	Yes	Yes	Yes	Yes	Yes
Provider of 1st check	X	Yes	Yes	Yes	Yes	Yes	Yes	Yes
Place of 1st check	X	Yes	Yes	Yes	No	No	Yes	Yes
**Postnatal care: Newborn**								
Timing of 1st check	X	X	X	Yes	Yes	Yes	Yes	Yes
Provider of 1st check	X	X	X	Yes	Yes	Yes	Yes	Yes
Place of 1st check	X	X	X	Yes	Yes	Yes	Yes	Yes
Content of check	X	X	X	X	X	X	Yes	Yes

X – Information not collected in the survey

The current phase 7 questionnaires introduced since 2013, continue to ask PNC questions of all women and newborns, but with additional questions to increase accuracy. Examples of what constitute a health check was provided for both women and newborns. Each category of respondents (ie, women with facility delivery who were checked before discharge, those who were not checked while in facility, and those who delivered outside a facility) has a separate set of questions investigating postnatal care for mothers and newborns. For the first time in this round, women who delivered and were checked in a health facility before discharge are once again asked questions about any check on health, time, provider and location of health checks following discharge to capture a subsequent postnatal health check. Similarly, questions about any postnatal health check of the newborn after discharge are asked of women with a facility delivery. As a result, the current phase of DHS may provide data on an additional postnatal health check for facility births. Another substantial addition in this round of survey is questions on content of PNC for mother and newborn. All women, whether they delivered in or outside a health facility are asked if a health care provider examined the cord, measured temperature, counseled on danger signs, and observed breastfeeding within two days of birth of the baby.

### Measuring PNC in MICS

**Table 3** describes measurement of PNC in MICS questionnaires. MICS introduced standard PNC questions in a module referred to as “Postnatal Health Checks” during the fourth round of the survey starting from 2009, although there were few prior surveys that had included limited PNC questions based on countries’ specific initiatives. The MICS4 module collected detailed information on postnatal health contacts after delivery and distinguished an immediate health check from a postnatal visit for all mothers and births regardless of place of delivery. The module was developed following consultation with the Newborn Indicators Technical Working Group, coordinated by Save the Children. Since MICS4, the PNC modules have been fairly consistent for both women and newborn. The latest round (MICS 7), initiated from 2017, introduced questions on PNC content, similar to the DHS questionnaires.

**Table 3:** Overview of postnatal care (PNC) data collected in Multiple Indicator Cluster Surveys (MICS)

Postnatal care: women & Newborn	MICS 1–3 (1993–2009)	MICS 4 (2009–2013)			MICS 5 (2013–2016)			MICS 6 (2017–ongoing)		
	**Facility births**	**Assisted home births**	**Unassisted home births**	**Facility births**	**Assisted home births**	**Unassisted home births**	**Facility births**	**Assisted home births**	**Unassisted home births**
**Immediate check:**										
Time of check	X	No	No	X	No	No	X	No	No	X
Provider of check	X	No	No	X	No	No	X	No	No	X
Place of check	X	Yes	Yes	X	Yes	Yes	X	Yes	Yes	X
**Postnatal visit:**										
Time of 1st visit	X	Yes	Yes	Yes	Yes	Yes	Yes	Yes	Yes	Yes
Place of 1st visit	X	Yes	Yes	Yes	Yes	Yes	Yes	Yes	Yes	Yes
Provider of 1st visit	X	Yes	Yes	Yes	Yes	Yes	Yes	Yes	Yes	Yes
Content of PNC visit	X	X	X	X	X	X	X	Yes	Yes	Yes

X – Information not collected in the survey

### Implications for calculation of the global PNC indicator

Differences in the PNC questions between the two surveys programs have led to differences in the methodological approach used to compute the coverage of the PNC within two days after delivery, a potential source of incomparability (**Table 4**). To calculate this indicator, MICS distinguishes immediate check from PNC visits (post discharge or after attendant left in case of non–institutional deliveries) for mothers and newborns up to two days after delivery. A woman or newborn is then considered as having received PNC within two days after birth if an immediate check or a PNC visit occurred within these two days. DHS on the other hand, does not make this distinction and includes only the first health check after delivery that may occur anytime between birth and two days following delivery, regardless of whether it was the immediate check or the later PNC visit (see Figure S1 and S2 in **Online Supplementary Document(Online Supplementary Document)**). However, from phase 7 onwards DHS, questions separating out immediate check (pre–discharge check) from a later postnatal visit (post–discharge check) were introduced.

**Table 4:** Comparison of postnatal care (PNC) indicator measured in Demographic and Health Surveys (DHS) phase 6 and Multiple Indicator Cluster Surveys (MICS) round 4–6 questionnaires

Data collected	DHS	MICS
**Respondents:**		
	Institutional births	Institutional births
	Non–institutional births	Non–institutional births with attendants
		Non–institutional births without attendants
**Global indicator for postnatal care for mothers:**		
Numerator	Number of women aged 15–49 years who received a health check within 2 days after delivery	Number of women aged 15–49 years who received a health check while in facility or at home following delivery, or a post–natal care visit within 2 days after delivery
Denominator	Total number of women aged 15–49 years with a live birth in the 2 years preceding the survey **(DHS changed reference period from five to two years)**	Total number of women aged 15–49 years with a live birth in the 2 years preceding the survey
**Global indicator for postnatal care for newborns:**		
Numerator	Number of last live births in the last 2 years who received a health check within 2 days after birth	Number of last live births in the last 2 years who received a health check while in facility or at home following delivery, or a post–natal care visit within 2 days after birth
Denominator	Total number of last live births in the last 2 years **(DHS changed reference period from five to two years)**	Total number of last live births in the last 2 years
**First PNC contact**	Unable to differentiate immediate health check from later postnatal visit	Able to distinctly assess and measure immediate health check from postnatal visit
Provider of first check	Yes	No, implied for institutional deliveries
Place of first check	Yes	Yes
Timing of first check	Yes	No
**Duration of stay in facility**	Yes	Yes
**Postnatal visit**	No	Yes, including timing, location and provider of first PNC visit
**Content of PNC**	Yes (starting in Phase 7)	Yes, starting in round 6

Assessment of the questionnaires from MICS 4 or 5 and DHS phase 6 further reveals that though the wording of questions about postnatal care of women are fairly similar across the two survey programs such as providing examples of a health check, a fundamental difference exists in the way questions are framed for postnatal care of newborns. While MICS asks about immediate health check and postnatal visits for the newborn without a specific reference period, DHS’ questions on PNC for newborns considers checks within two months following the birth of the baby. However, in the most recent versions of the DHS (DHS phase 7) and MICS questionnaires, questions about postnatal care for women and newborns are closely aligned for institutional births but remain inconsistent for non–institutional births.

### Comparing PNC coverage and timing between surveys

**Table 5** and **Table 6** show measures of PNC respectively for women and newborns, comparing MICS and DHS results in the four countries. The pattern explained above, of broadly similar coverage of PNC for women between the two types of surveys and largely different coverage of PNC for newborns is seen in these four countries. Using MICS data, which allows us to distinguish between immediate (pre–discharge) checks and (post–discharge) postnatal visits, we see a higher proportion of newborns receiving a postnatal visit compared to women, while coverage of the immediate check is not very dissimilar for women and newborns.

**Table 5:** Postnatal care for women across Demographic and Health Surveys (DHS) and Multiple Indicator Cluster Surveys (MICS)

	Bangladesh		Ghana		Kyrgyzstan		Nepal		
**Women with non–institutional births:**									
	**DHS** **2014**	**MICS 2012–13**	**DHS** **2014**	**MICS** **2011**	**DHS** **2012**	**MICS** **2014**	**DHS** **2011**		**MICS** **2014**
n	**1932**	**5391**	**572**	**825**	**5**	**11**	**1143**		**894**
Any postnatal care	46.9	26.2	53.7	57.1	NR		13.7		20.6
Immediate health check	NA	22.1	NA	47.2	NA		NA		16.3
Postnatal visit	NA	8.7	NA	21.9	NA		NA		7.2
Postnatal health check within 2 d of delivery	42.4	24.5	44.9	50.9	NR		11.3		18.4
**Women with institutional births:**									
n	**1272**	**2461**	**1691**	**1703**	**1686**	**1648**	**888**	**1130**	
Any postnatal care	92.7	78.4	95.9	97.7	98.5	99.2	88.5	90.6	
Immediate health check	92.2	76.5	95.2	97.5	98.3	99.1	87.8	90.5	
Postnatal visit	NA	17.8	NA	26.6	NA	55.6	NA	17.0	
Postnatal health check within 2 days of delivery	86.8	76.9	93.4	97.5	96.3	99.1	87.3	90.6	
All women (check within 2 days)	60.1	40.4	81.2	82.3	95.9	97.8	44.5	57.9	

NA – question not asked, NR – analysis not reported due to small sample size

**Table 6:** Postnatal care for newborn across Demographic and Health Surveys (DHS) and Multiple Indicator Cluster Surveys (MICS)

	Bangladesh		Ghana		Kyrgyzstan		Nepal		
**Non–institutional births:**									
	**DHS 2014**	**MICS 2012–13**	**DHS 2014**	**MICS 2011**	**DHS 2012**	**MICS 2014**	**DHS 2011**		**MICS 2014**
n	**1932**	**5391**	**572**	**825**	**5**	**11**	**1143**		**894**
Any postnatal care	51.7	25.9	66.6	62.3	48.8	59.6	30.0		22.1
Immediate health check	NA	20.9	NA	47.4	NA	29.8	NA		15.0
Postnatal visit	NA	11.3	NA	35.9	NA	54.6	NA		11.7
Postnatal health check within 2 days of delivery	41.5	23.8	16.5	54.0	48.8	59.6	9.6		17.6
**Institutional births:**									
n	**1272**	**2461**	**1691**	**1703**	**1686**	**1648**	**888**	**1130**	
Any postnatal care	81.7	83.3	73.4	97.9	89.5	99.9	68.5	91.2	
Immediate health check	NA	80.5	NA	97.1	NA	99.7	NA	90.6	
Postnatal visit	NA	26.6	NA	44.3	NA	94.9	NA	22.3	
Postnatal health check within 2 days of delivery	74.3	80.9	24.9	97.1	80.0	99.7	56.5	90.6	
All births (check within 2 days)	54.5	41.2	22.8	83.1	79.8	98.5	30.1	57.7	

NA – question not asked, NR – analysis not reported due to small sample size

**Figure 2:**
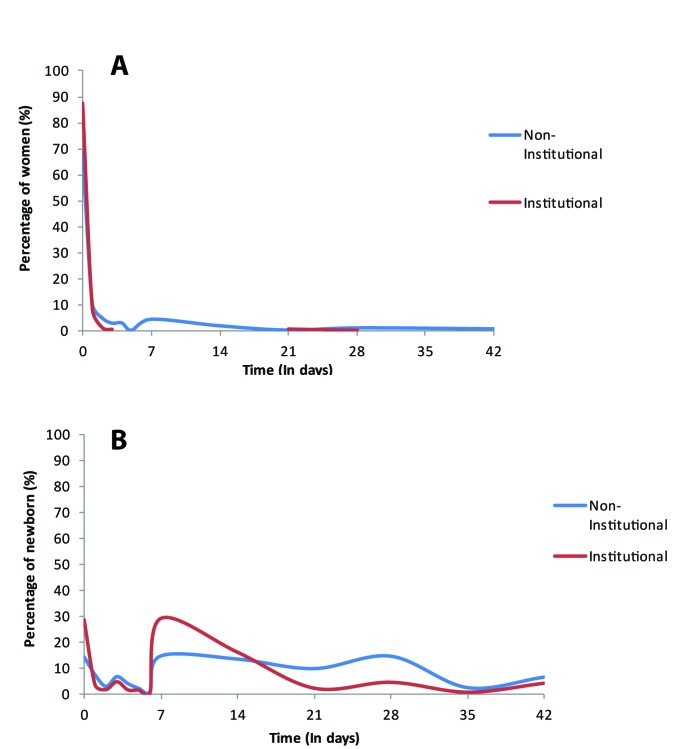
Timing of health check, Ghana Demographic and Health Surveys (DHS) 2014. **A.** Women. **B.** Newborn.

**Figure 3:**
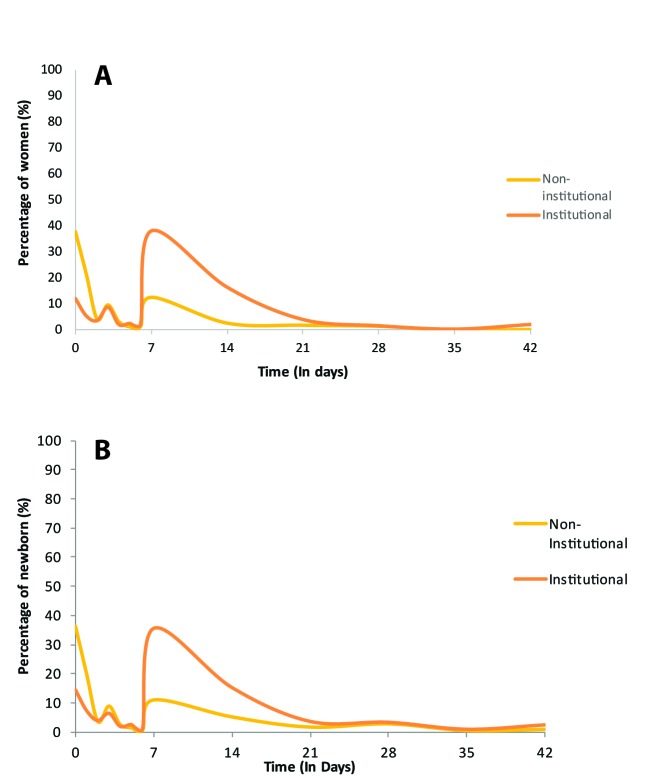
Timing of postnatal visit, Ghana Multiple Indicator Cluster Surveys (MICS) 2011. **A.** Women. **B.** Newborn.

In **Figure 2** and **Figure 3**, we assess the distribution of timing of health check for women and newborn in Ghana, respectively for DHS and MICS. From the DHS, most women appear to report a health check for themselves within the first days following delivery, most of the first day for institutional deliveries. However for their newborn, the reported coverage is much lower overall and the bimodal distribution (at day 0 and day 7) indicates that a substantial number of women report PNC on day 7 rather than within the first two days. For MICS, the figures include only the distribution of timing of the post–discharge or postnatal visit for women and newborn as the survey does not collect information on timing of immediate health check. The distribution of timing of postnatal visit is nearly identical for the mother and the newborn regardless of place of delivery.

## DISCUSSION

Postnatal care is one of the essential strategies recommended for scale–up in many countries to improve health outcomes for women and newborn. The proportion of women receiving postnatal care within two days of delivery and the proportion of newborns receiving postnatal care within two days of delivery are the global consensus indicators for monitoring the coverage of this practice by countries. While enormous progress has been made in the past decade to accurately measure these indicators through household surveys, monitoring of levels and trend require consistent measurement across survey programs, time and geographies. We reviewed the way data on PNC indicators have been collected and the methodology used for their computation focusing on the two largest household survey programs, MICS and DHS. Results showed that the two survey programs have not measured the PNC indicator consistently, both in the way the questions are framed and the approach used for computation of this indicator. MICS dedicated a detailed standalone module to collect information on PNC for mother and newborn and included details that try to capture immediate checks following delivery from subsequent postnatal visit following discharge from health facility (in case of facility delivery) or when the attendant has left (in case of out–of facility deliveries with health professional or trained birth attendant). Their approach and questions used are similar for mother and newborns. The calculation approach of the PNC indicators for either mothers or newborns thus captures occurrence of an immediate and/or a later postnatal visit occurring within the two–days window. By segmenting the postnatal period, this approach aims to better trigger the memory of the respondent toward a more accurate response. Consequently, coverage levels based on this approach tend to be higher than that of the DHS, and similar for both women and newborns.

DHS, on the other hand, implements a blended pregnancy and PNC module and measures the indicator differently for mothers and newborns. It does not systematically distinguish the immediate vs postnatal visit. Furthermore, for newborns, PNC questions in DHS reference to a check in the two month period following birth. The resulting coverage measures show a much lower PNC rate for newborns compared to women. We suspect that when mothers are asked during interviews about PNC of their newborn within the two months following the delivery, they are more likely to recall the most recent visit, which is likely to fall outside the first two days, resulting in an under–estimation of the coverage indicator. We indeed found a divergence in the distribution of timing of postnatal checks in DHS, with women reporting most immediate care for themselves (thus resulting in high coverage of PNC) while for their newborns a substantial number of women tend to report the check at day 7, resulting in lower coverage of PNC within two–day of delivery for newborns. Because such two–month reference window is not applied to women themselves, they are likely to report more accurately a check that occurred within two days of delivery, especially given pre–discharge questions were specified. However, the confusion between when the intrapartum period ends and the postnatal period begins means that the immediate health check may also be capturing immediate intrapartum checks not necessarily considered as postnatal health check. A qualitative study in Malawi and Bangladesh suggested that women may potentially be reporting on a routine intrapartum check rather than a distinct postnatal contact [15,16]. This may result in overestimation of PNC measures in women for DHS and in both women and newborns for MICS.

While our analysis does not constitute a validation of one approach vs the other, a clear and most actionable implication is for MICS and DHS to coordinate and align the measurement of such critical indicators to improve comparability between measures coming from the two survey programs. Until such alignment occurs, measures produced will not be comparable. There are also increasing calls for going beyond measures of contact such as PNC to incorporate measure of content interventions received by mothers and newborns during these contacts [26,27]. A simple measure of contact does not provide any indication of the quality of care received, the duration and contents of such contacts. The most recent MICS round 6 and DHS phase 7 have both included a number of questions on the content of the first check within the first 2 days following birth, including cord examination, weight and temperature assessment, breastfeeding counseling and observation and counseling on symtpoms that cause a mother to take a newborn to health care. These efforts must also be guided by clear recommendation from the maternal and newborn community on content of PNC and its quality. The recent revisions to the DHS and MICS tools to improve alignment in the measurement of PNC indicators and incorporate information on PNC content is a welcome step toward filling these data gaps. However, the currents tools are still not fully aligned on PNC measurement, especially for out–of–facility deliveries.

Furthermore, more studies must to be carried out to validate reports from mothers on intrapartum and postpartum care during household survey to help fine tune the measurement tools.
