# Supplementary material for: Measuring postnatal care contacts for mothers and newborns: An analysis of data from the MICS and DHS surveys
Source: J Glob Health. 2017 Dec 20;7(2):020502. doi: 10.7189/jogh.07.020502 (PMC5804502; doi:10.7189/jogh.07.020502)
Supplement: Online Supplementary Document [file jogh-07-020502-s001.pdf]

Online Supplementary Document

Amouzou et al. Measuring postnatal care contacts for mothers and newborns: An analysis of data from the MICS and DHS surveys

J Glob Health 2017;7:020502

A. Additional Results

Figure S1: Algorithm of Postnatal Health Checks in DHS phase 6

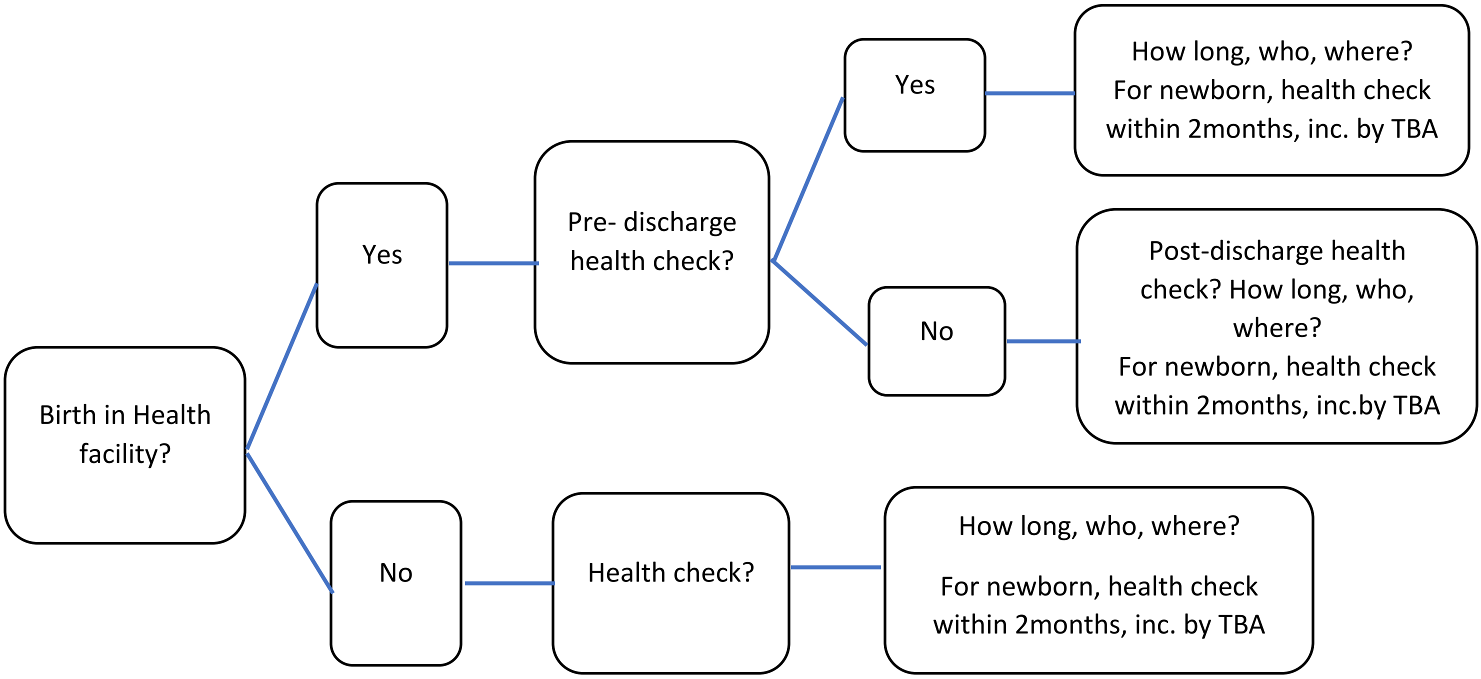

Figure S2: Algorithm of Postnatal Health Checks in MICS 5

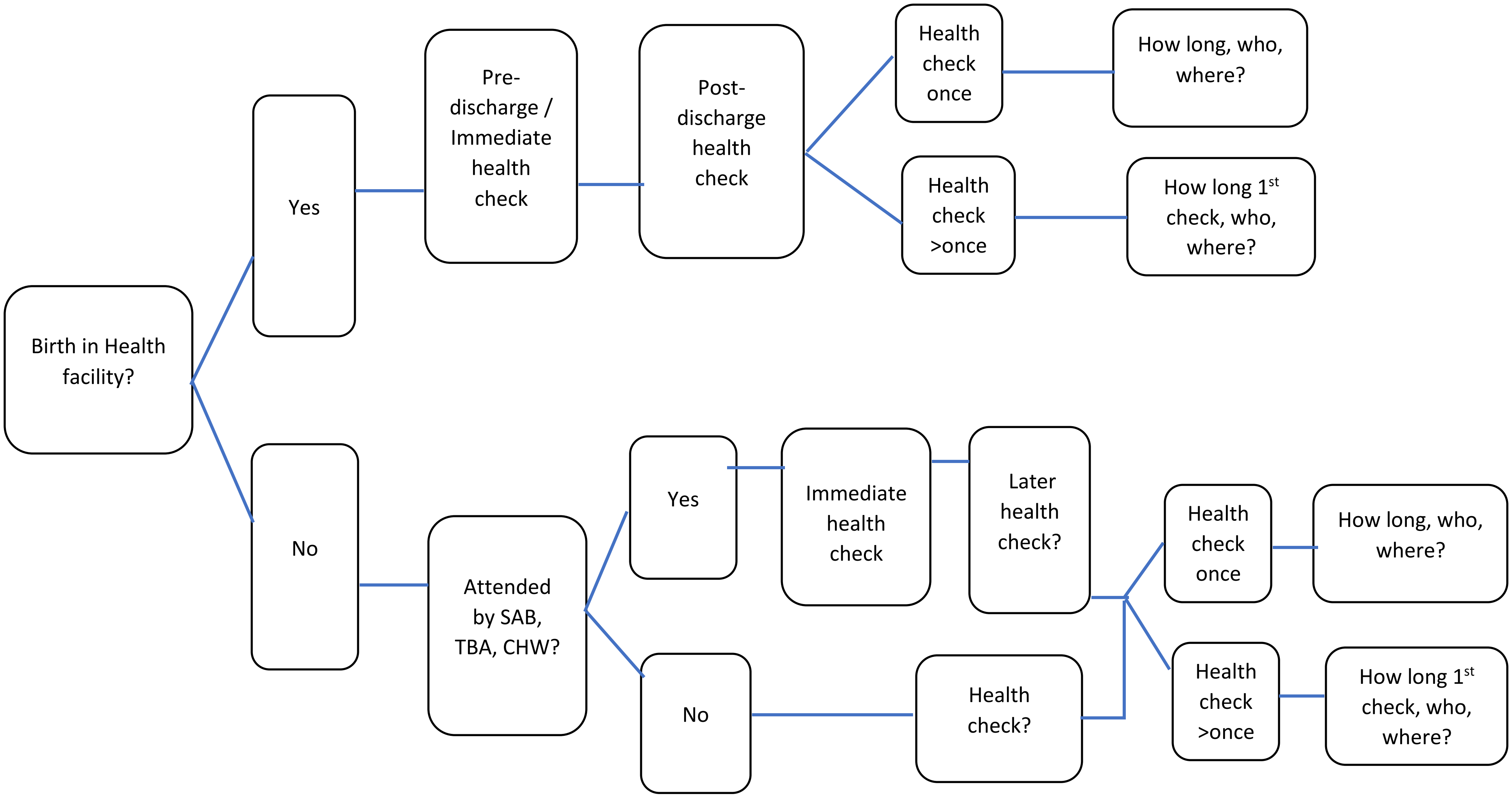

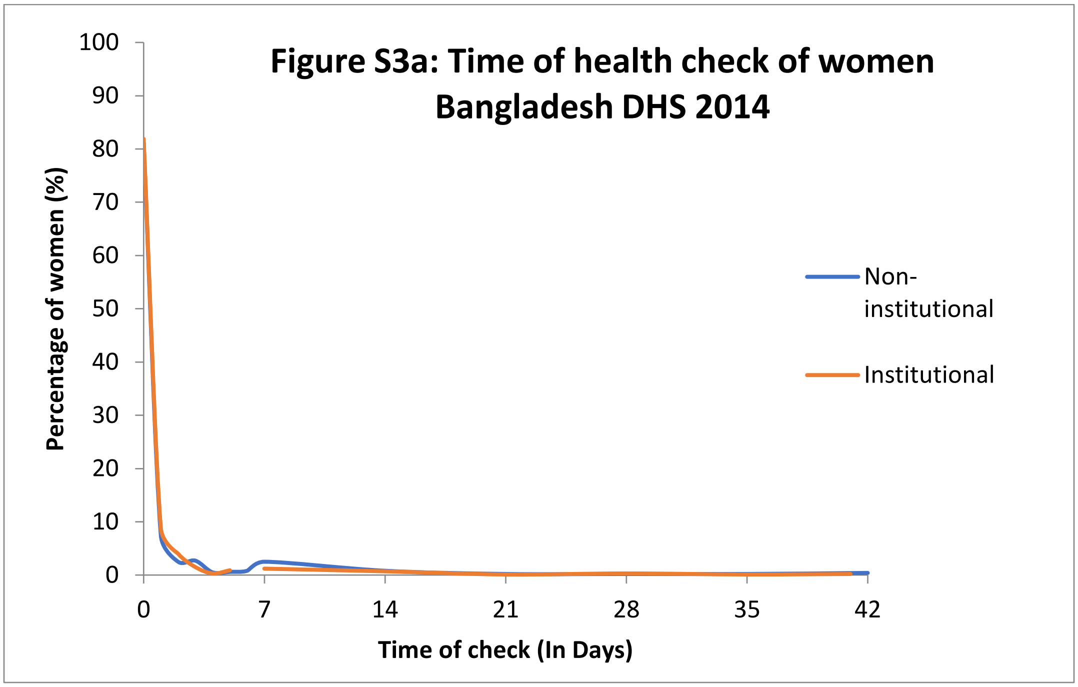
Figure S3a: Time of health check of women
Bangladesh DHS 2014
Percentage of women (%)
100
90
80
70
60
50
40
30
20
10
0
0
7
14
21
28
35
42
Time of check (In Days)
Non-institutional
Institutional

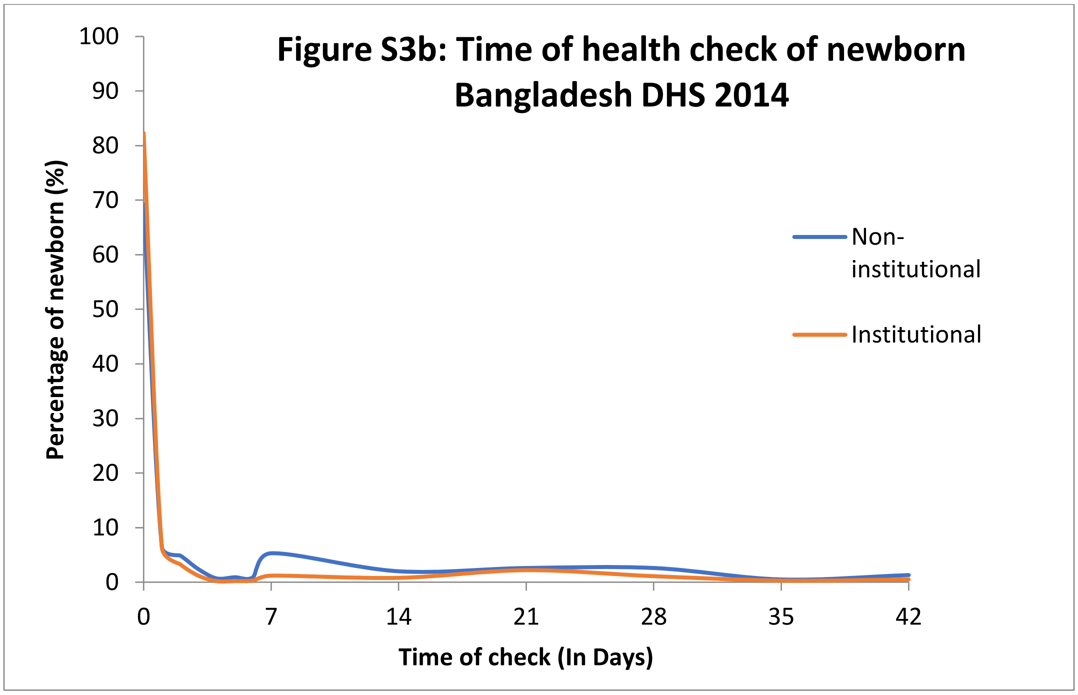
Figure S3b: Time of health check of newborn
Bangladesh DHS 2014
Percentage of newborn (%)
100
90
80
70
60
50
40
30
20
10
0
0
7
14
21
28
35
42
Time of check (In Days)
Non-institutional
Institutional

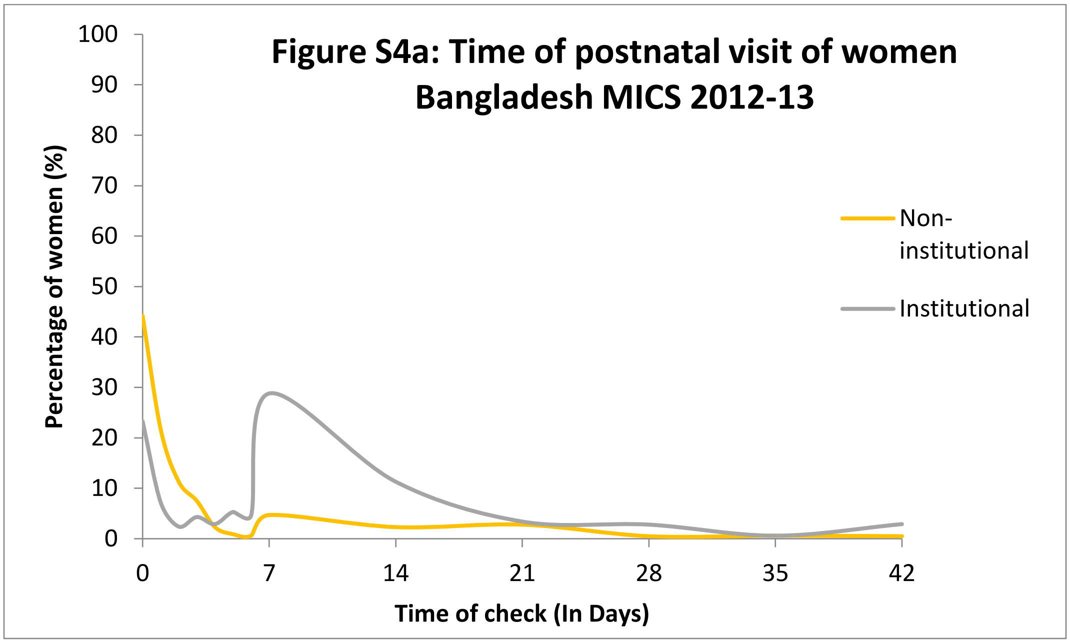
Figure S4a: Time of postnatal visit of women
Bangladesh MICS 2012-13
100
90
80
70
60
50
40
30
20
10
0
Percentage of women (%)
0
7
14
21
28
35
42
Time of check (In Days)
Non-institutional
Institutional

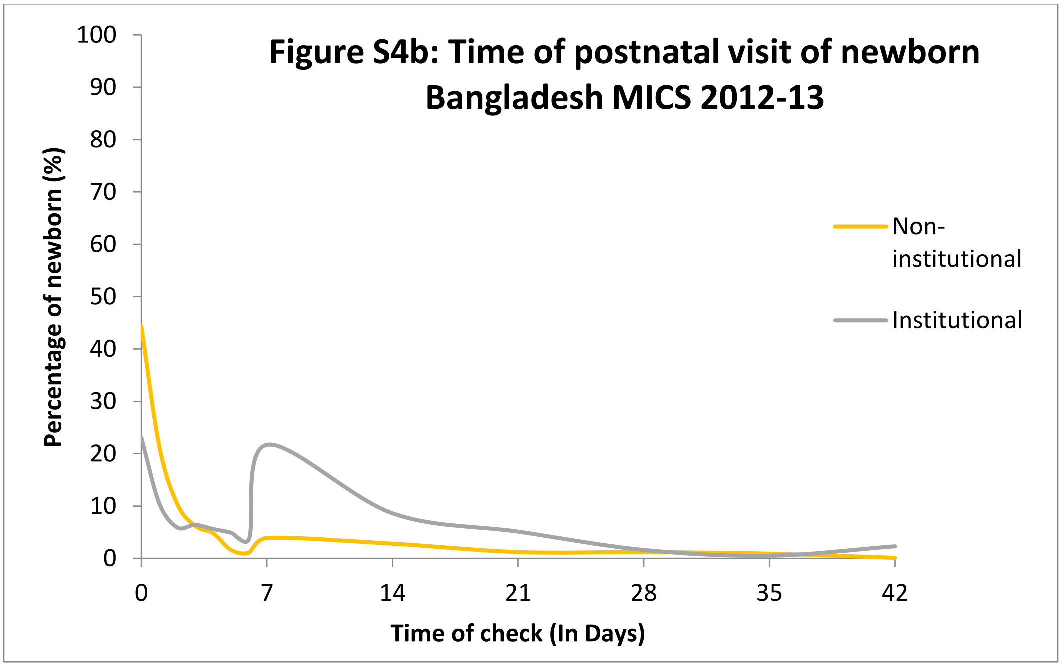
Figure S4b: Time of postnatal visit of newborn
Bangladesh MICS 2012-13
100
90
80
70
60
50
40
30
20
10
0
Percentage of newborn (%)
0
7
14
21
28
35
42
Time of check (In Days)
Non-institutional
Institutional

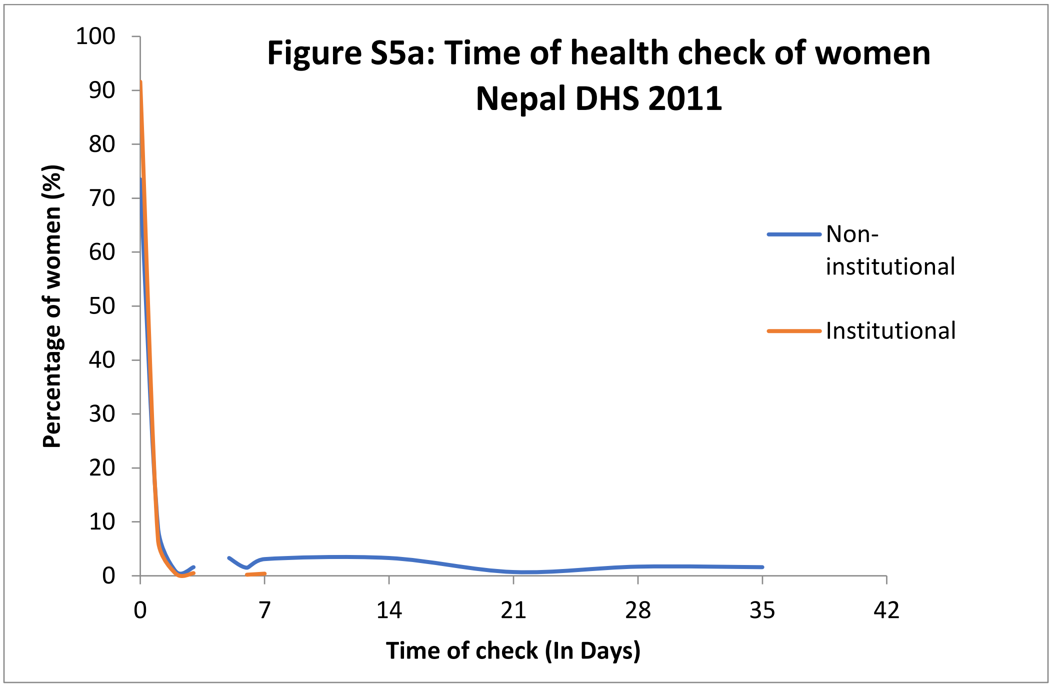
Figure S5a: Time of health check of women
Nepal DHS 2011
Percentage of women (%)
100
90
80
70
60
50
40
30
20
10
0
0
7
14
21
28
35
42
Time of check (In Days)
Non-institutional
Institutional

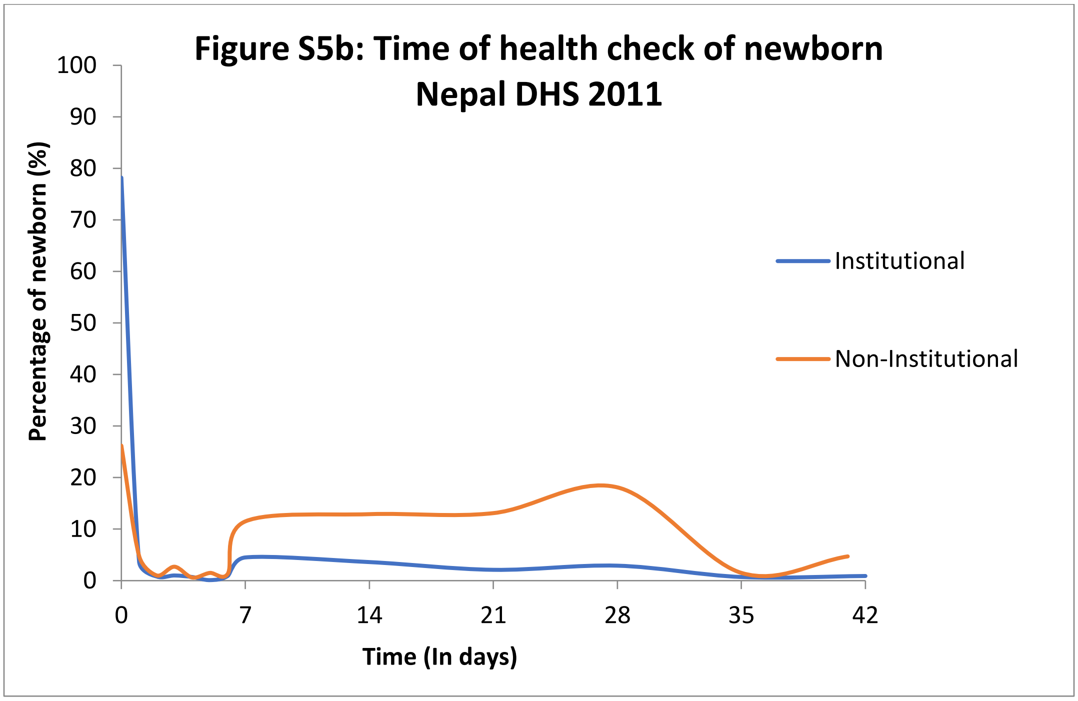
Figure S5b: Time of health check of newborn
Nepal DHS 2011
Percentage of newborn (%)
100
90
80
70
60
50
40
30
20
10
0
0
7
14
21
28
35
42
Time (In days)
Institutional
Non-Institutional

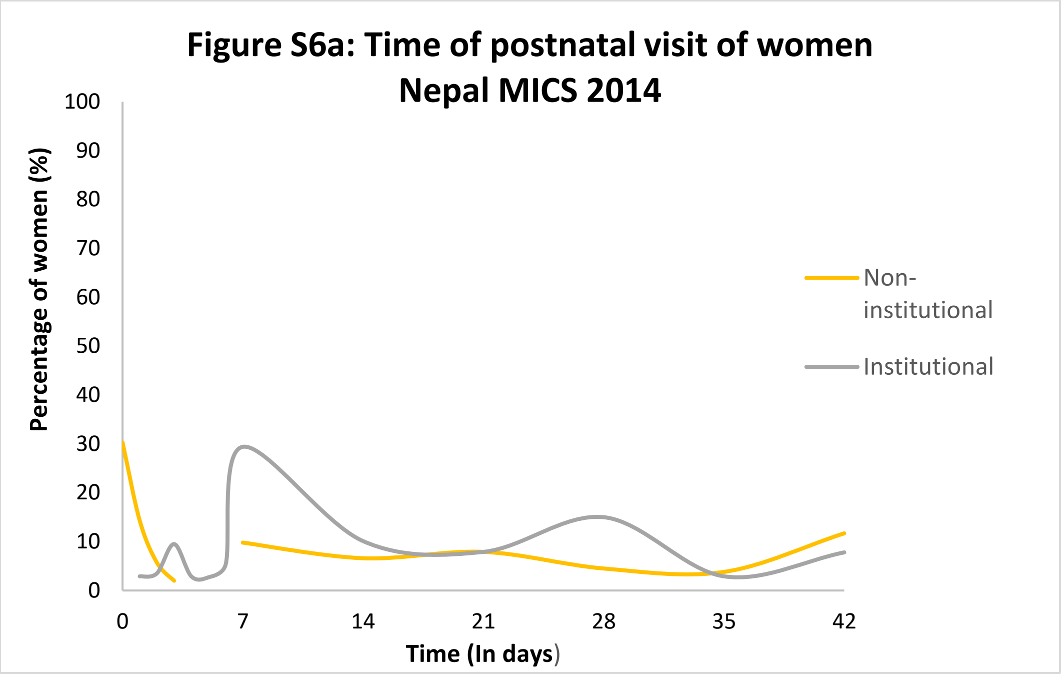
Figure S6a: Time of postnatal visit of women
Nepal MICS 2014
Percentage of women (%)
100
90
80
70
60
50
40
30
20
10
0
0
7
14
21
28
35
42
Time (In days)
Non-institutional
Institutional

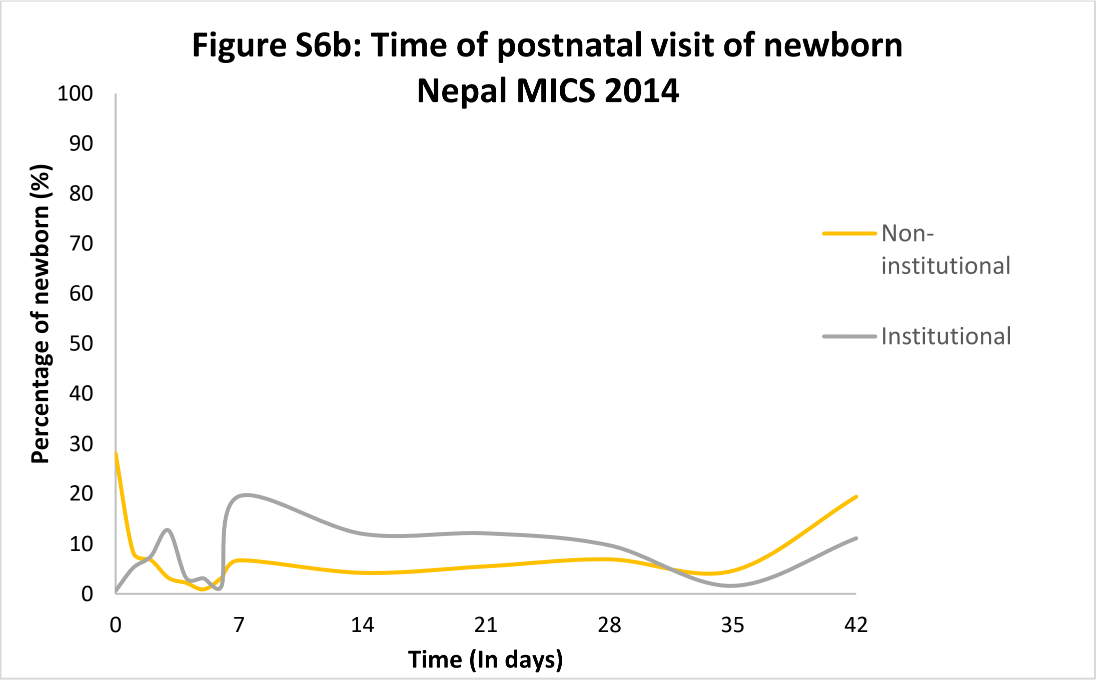
Figure S6b: Time of postnatal visit of newborn
Nepal MICS 2014
Percentage of newborn (%)
100
90
80
70
60
50
40
30
20
10
0
0
7
14
21
28
35
42
Time (In days)
Non-institutional
Institutional

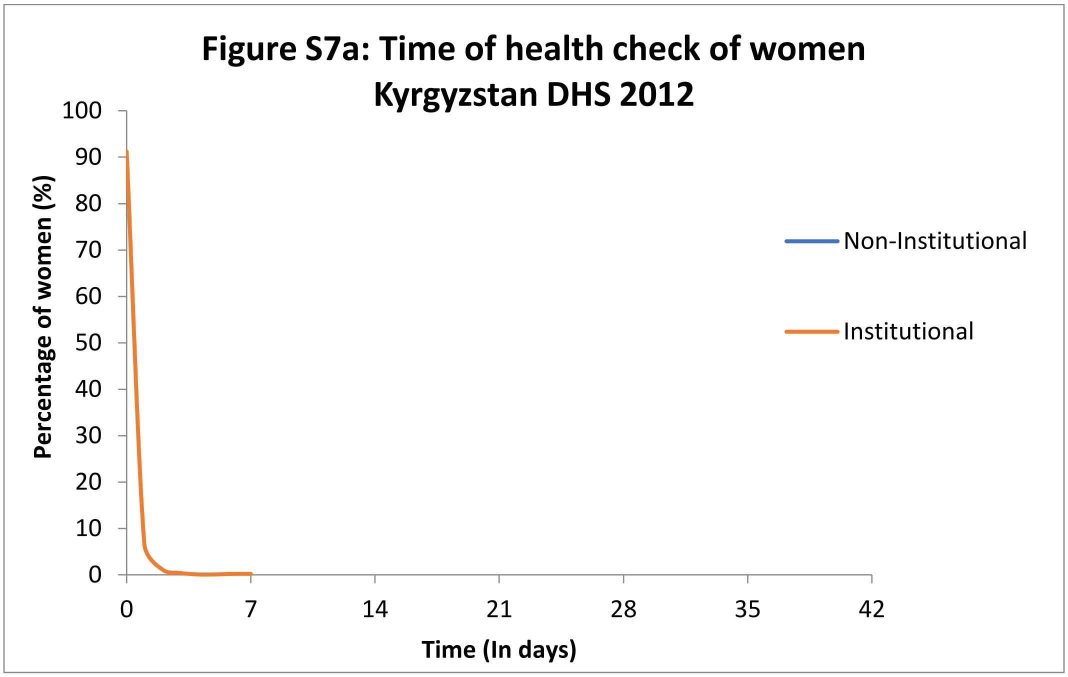

Figure S7a: Time of health check of women
Kyrgyzstan DHS 2012
Percentage of women (%)
100
90
80
70
60
50
40
30
20
10
0
0
7
14
21
28
35
42
Time (In days)
Non-Institutional
Institutional

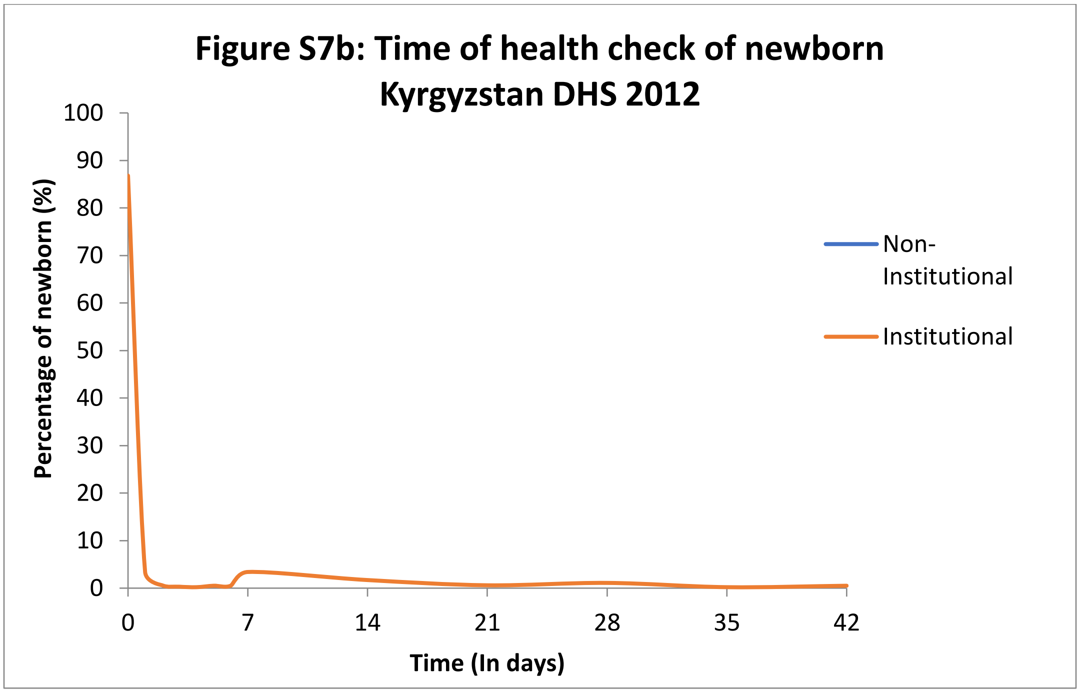

Figure S7b: Time of health check of newborn
Kyrgyzstan DHS 2012
Percentage of newborn (%)
100
90
80
70
60
50
40
30
20
10
0
0
7
14
21
28
35
42
Time (In days)
Non-Institutional
Institutional

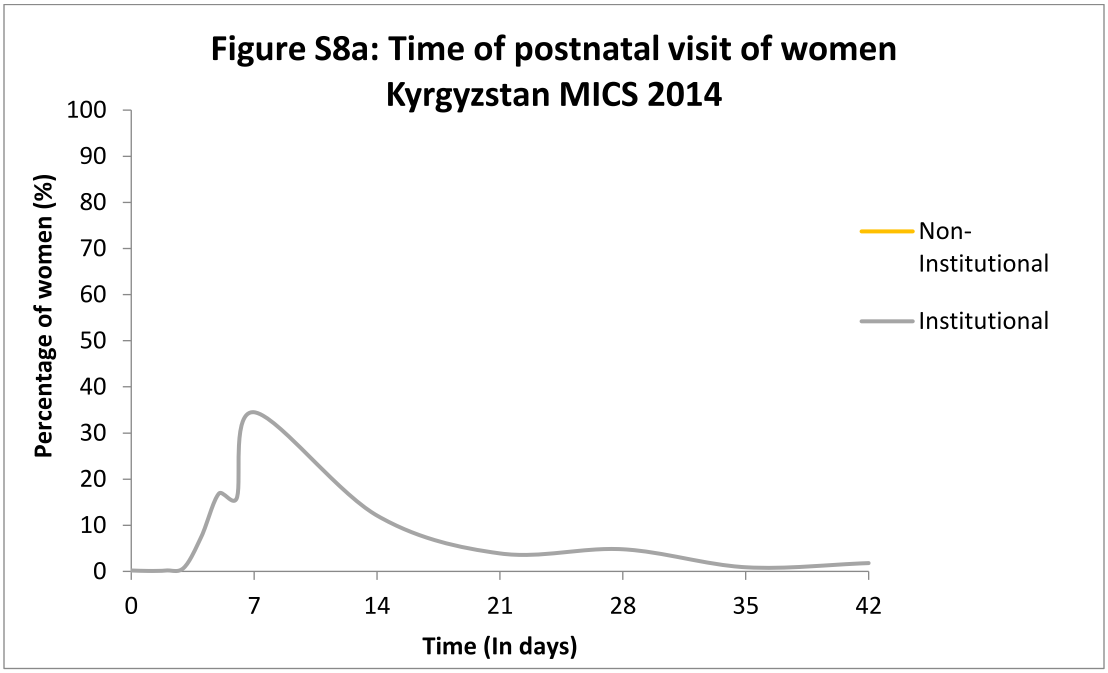
Figure S8a: Time of postnatal visit of women
Kyrgyzstan MICS 2014
Percentage of women (%)
100
90
80
70
60
50
40
30
20
10
0
0
7
14
21
28
35
42
Time (In days)
Non-Institutional
Institutional

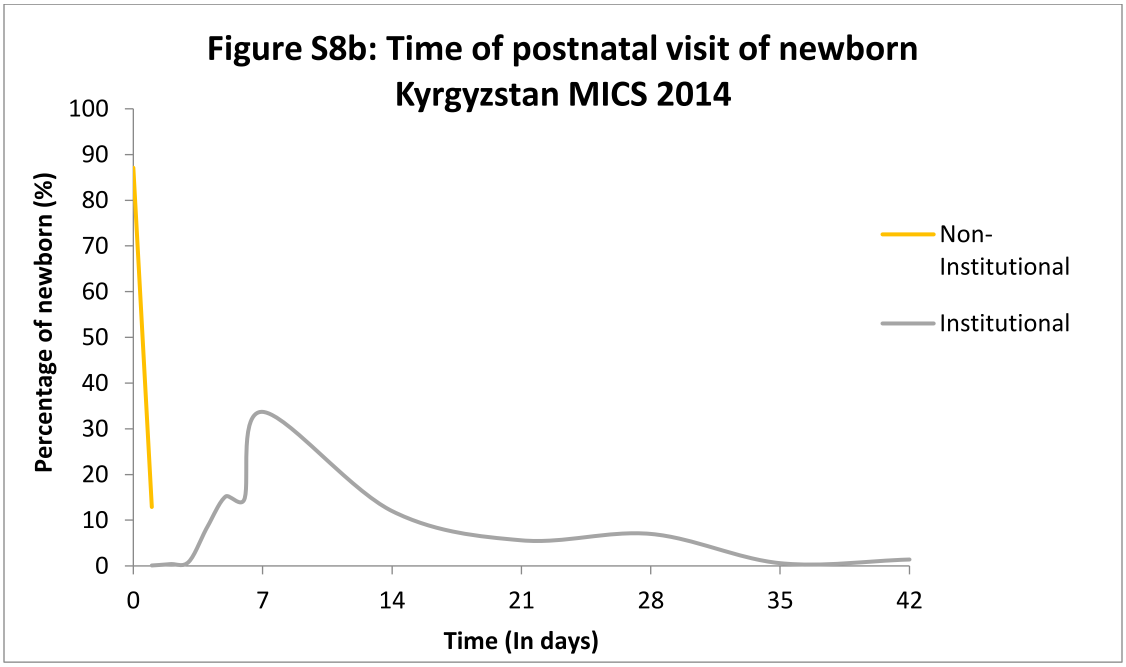
Figure S8b: Time of postnatal visit of newborn
Kyrgyzstan MICS 2014
Percentage of newborn (%)
100
90
80
70
60
50
40
30
20
10
0
0
7
14
21
28
35
42
Time (In days)
Non-Institutional
Institutional
